# Force-based control strategy for a collaborative robotic camera holder in laparoscopic surgery using pivoting motion

**DOI:** 10.3389/frobt.2023.1145265

**Published:** 2023-04-17

**Authors:** Carlos Fontúrbel, Ana Cisnal, Juan Carlos Fraile-Marinero, Javier Pérez-Turiel

**Affiliations:** Escuela de Ingenierías Industriales, Medical Robotics Group, Instituto de las Tecnologías Avanzadas de la Producción (ITAP), Universidad de Valladolid, Valladolid, Spain

**Keywords:** robotic surgery, laparoscopy, force control, collaborative robotics, admittance control

## Abstract

**Introduction:** Laparoscopic surgery often relies on a fixed Remote Center of Motion (RCM) for robot mobility control, which assumes that the patient’s abdominal walls are immobile. However, this assumption is inaccurate, especially in collaborative surgical environments. In this paper, we present a force-based strategy for the mobility of a robotic camera-holder system for laparoscopic surgery based on a pivoting motion. This strategy re-conceptualizes the conventional mobility control paradigm of surgical robotics.

**Methods:** The proposed strategy involves direct control of the Tool Center Point’s (TCP) position and orientation without any constraints associated with the spatial position of the incision. It is based on pivoting motions to minimize contact forces between the abdominal walls and the laparoscope. The control directly relates the measured force and angular velocity of the laparoscope, resulting in the reallocation of the trocar, whose position becomes a consequence of the natural accommodation allowed by this pivoting.

**Results:** The effectiveness and safety of the proposed control were evaluated through a series of experiments. The experiments showed that the control was able to minimize an external force of 9 N to ±0.2 N in 0.7 s and reduce it to 2 N in just 0.3 s. Furthermore, the camera was able to track a region of interest by displacing the TCP as desired, leveraging the strategy’s property that dynamically constrains its orientation.

**Discussion:** The proposed control strategy has proven to be effective minimizing the risk caused by sudden high forces resulting from accidents and maintaining the field of view despite any movements in the surgical environment, such as physiological movements of the patient or undesired movements of other surgical instruments. This control strategy can be implemented for laparoscopic robots without mechanical RCMs, as well as commercial collaborative robots, thereby improving the safety of surgical interventions in collaborative environments.

## 1 Introduction

Laparoscopic surgery is a widely accepted and extended technique in the current medical context. In this type of interventions, small incisions are made in the patient’s abdominal area. Through these open orifices, laparoscopic forceps, needle holders, laparoscope, and any other surgical tools are introduced. This minimally invasive surgical technique is beneficial for both surgeons and patients, leading to a postoperative recovery with fewer complications (Rau and Hünerbein, 2005).

The da Vinci is the market leader in robotic surgery. This robotic system is capable of performing complete laparoscopic surgeries under surgeon’s control. However, the large investment required in infrastructure and its high cost make it unaffordable for a large number of hospitals (Crew, 2020). One cost-effective solution is to integrate partially automated surgical systems, such as robotic assistants for performing individual tasks (Voros et al., 2010). For example, the laparoscope handling could be automated, mitigating human errors and saving resources, since it is currently handled by a medical assistant. Robotic solutions for assistance in laparoscopy surgery, such as laparoscope handling, have been proposed in the literature since the end of the 20th century (Hurteau et al., 1994), (Taylor et al., 1995). However, their integration into such collaborative systems remains a challenge due to the complexity of the human–robot interaction (Haddadin, 2014). In addition to the price, another limitation of the da Vinci is its absence of force feedback, which makes it difficult to control the contact forces at the incision.

Nowadays, the most widespread mobility method of robotic systems in the context of laparoscopy surgery is based on a remote centre of motion (RCM), coinciding with the entry point of the tool through the incision. It simplifies the development and control of robotic laparoscopic assistants (Voros et al., 2010; Kuo et al., 2012; Voros et al., 2010). There are strategies for the RCM implementation based on the mechanical design, as in the da Vinci (Freschi et al., 2013) or in Aksungur (2015) and Zhou et al. (2018), or based on control (Sandoval et al., 2021), where a virtual RCM is defined at the start of the surgery. These robots that include the RCM as a central feature of their design are based on the immobility of the entry point, thus defining the mobility constraints of the robot.

The premise of the staticity of the RCM approach omits the actual movement due to the physiological activity of the patient, such as the patient’s abdomen ventilation, which causes movement at the incision point (Valenza et al., 2010). Additionally, the trocar’s rest position varies for each patient. It is mainly affected by the patient’s abdominal elasticity coefficient, which depends on fat and other body parameters. This cannot be taken lightly in collaborative environments, where accidents can occur, or larger forces can be exerted by the surgical instruments that displace the patient’s abdomen. Control strategies have been proposed to reduce the force exerted at the contact point between the trocar and the patient by measuring this force. This approach was tested in the early 21st century by Krupa et al. (2000), implementing what is now considered a mobile RCM (Marinho et al., 2016). The results were not fully satisfactory due to the low frequency of the force sensor, which made a truly effective control infeasible. This may be the reason why these force-based control strategies which ensures low contact forces are not widely extended in real surgical settings nowadays. Due to the improvement in technology, some hands-on robot approaches have appeared (Kastritsi and Doulgeri, 2021; Kim et al., 2021). They use a force sensor to move the robot to the desired position but always using the RCM as a fundamental mobility restriction.

In contrast to the traditional or mobile RCM-based solutions, this paper presents a mobility strategy based on pivoting the laparoscope to minimize the efforts measured by a force/torque sensor located in the coupling of the tool. The measured force is used to pivot the camera, minimizing the contact force without losing control of the camera’s TCP. This approach allows to perform an automatic control of the orientation of the tool, thus releasing the TCP control position. In this way, a pivoting motion is achieved, similar to the proposal made by Muñoz et al. (2005), with the advantages of having rigid joints and the use of a commercial robot. It allows to keep the desired field of view (FoV) using modern mobility and camera tracking strategies as proposed by Sandoval et al. (2021), with a greater degree of security since the contact forces are minimized in the background. The proposed control strategy has been tested for the handling of a laparoscope using a 6-DoF Universal Robots UR3e robot and a pelvitrainer. Trainee surgeons often use pelvitrainers as they allow them to practice individual aspects of minimally invasive surgical procedures, such as camera navigation or instrument handling, on a model rather than the patient (Ackermann et al., 2022). Tests have been carried out with different contact forces caused by the movement of a pelvitrainer or the laparoscope’s tip, thus simulating the patient’s physiological movement, or sudden accidental movements. In general terms, the control is able to minimize accidents in the operating room through rotations of the tool; it reduces the exerted forces at the trocar without losing the control of the laparoscope’s tip.

## 2 Materials and methods

### 2.1 Traditional RCM approach

**FIGURE 1:**
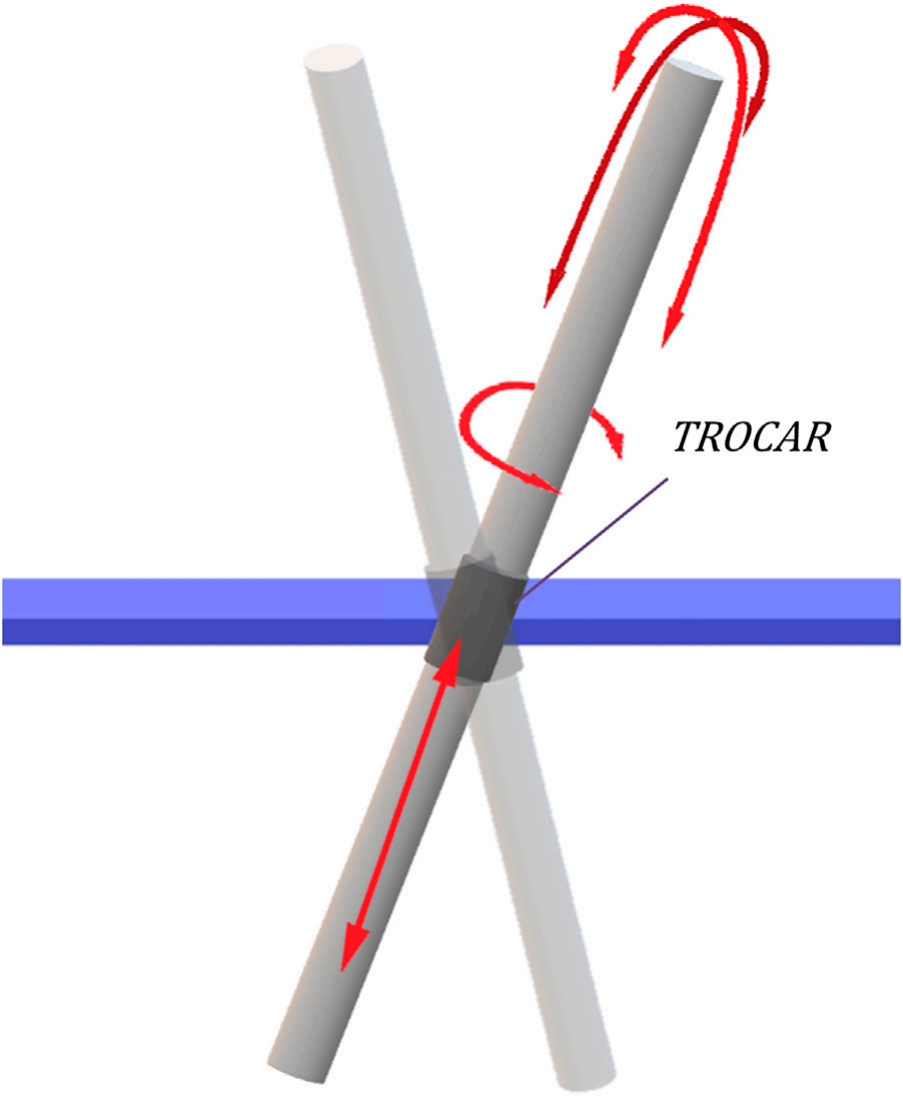
Four DoFs available using the RCM approach, which is coincident with the trocar position.

The traditional RCM approach considers the incision as a static point characterized by four DoFs: a translation along the axis of the instrument and three rotations around this axis (Figure 1). The surgical instrument penetrates the patient’s body through the incision point. The 4-DoF mobility constraints featured by the RCM generate a spherical working space for the TCP and can be mechanically imposed or virtually defined (Dombre et al., 2013). However, considering the incision as a fixed point that coincides with the RCM has some disadvantages. It requires a very precise positioning of the RCM with the incision point during surgery to avoid tissue damage (Dong and Morel, 2016) and also causes the Cartesian position of the TCP to be controlled indirectly.

Marinho et al. (2016) reported that it was possible to generate TCP linear movements through the implementation of a RCM by controlling the 4 available DoFs without an error in the RCM positioning. The primary goal of this approach is to keep the tool static at the RCM, preventing the occurrence of contact forces. Consequently, the TCP positioning is left as a secondary role in RCM-based implementations (Kim et al., 2021).

The popularity of the RCM approach, which considers the incision a static point, has limited the development of methods for minimizing the forces exerted at the trocar due to physiological movements of the patient or collaborative environments. Some methods that dynamically modify the position of the RCM by estimating the trocar position have been developed (Riviere et al., 2006; Dong and Morel, 2016; Richa et al., 2011). This approach, known as mobile RCM, causes difficulties in the camera repositioning as a result of the trocar’s movement during the intervention. If the exact application point of the force is unknown, the entire camera must perform a Cartesian motion in order to reduce the contact force of the trocar (Marinho et al., 2016). The RCM must coincide with the trocar to ensure that no force is exerted on the patient, since the position of the TCP is conditioned by the position of the RCM. To avoid this continuous movement, relatively high force thresholds must be applied, which limits the performance of this strategy. Hence, we considered that the mobile RCM is not an ideal solution once the non-staticity of the trocar is considered.

If the incision point is not considered static, the use of the RCM approach is no longer justified. If it is possible to measure the contact forces between the trocar and the patient, these forces should be able to be controlled independently of the TCP position, allowing precise independent control at the trocar and at the tip of the camera.

### 2.2 Proposed pivoting motion control

To overcome the disadvantages of the traditional approach based on a static RCM, a mobility strategy based on pivoting motion is presented. It consists of varying the orientation of the camera so as to minimize the contact forces with the abdominal walls of the patient. It adapts the laparoscope’s orientation to the real location of the incision, or in other words, to the trocar position at each moment. Since the laparoscope must keep track of the scenario and should not interfere with other instruments or tissues, we can infer that no other contact forces should affect the camera’s navigation. Therefore, an admittance control can determine the angular velocity (pivoting movement) of the camera required to minimize the contact forces, which are measured by a force/torque (F/T) sensor located in the coupling of the tool. In this way, the position of the TCP remains under direct control, while the pivoting motion minimizes the forces on the trocar, which always tends to a resting position.

This motion strategy is a restatement of the strategy proposed by Krupa et al. (2000), which considered that the position of the trocar varies over time. Although this control strategy was too complex to implement at the time, it is a valid approach today due to the improvements in sensorization in the last years. In this article, the absolute tool velocity was determined at the height of the trocar, displacing this point horizontally as a result of the force at the trocar. Krupa’s strategy was directly related to those that implement a mobile RCM, which have not been particularly fruitful in the literature. We consider that this approach, although it could not be widely extended due to technological deficiencies, as mentioned in the article itself, can be simplified by displacing the controlled point to the tip.

This force-based strategy allows to control the pivoting motion without the need of the passive joint. Multiple advantages associated with pivoting motion control have been previously reported in the literature (Muñoz et al., 2005). These advantages include, above all, repositioning the tool, in this case the camera, without losing control of its tip. Thus, it is possible to track the image even during the pivoting that may occur due to contact between the camera and patient. This allows lowering the threshold of admitted force before starting the reorientation, as well as solving the problem with a less aggressive movement, avoiding displacement of the entire camera and also through low magnitude turns.

In the following section, the admittance control will be introduced. It generates the pivoting motion of the tool to minimize the forces produced at the trocar. Second, the feedforward control is described, which avoids the occurrence of exerting forces due to the movement of the laparoscope. Third, the mobility constraints associated with the proposed control are addressed.

#### 2.2.1 Admittance control

The mechanical admittance (Y) of a system is defined as the ratio of its velocity (v) to its force (F), as expressed in Equation 1. This definition is related to the second-order differential equation which describes a mass, spring, and dumping serial system and hence, it is characterized by a mass (m), a stiffness (k), and a viscous damping (b) (Mihelj and Podobnik, 2012).

$$Y\left(s\right)=\frac{v}{F}=\frac{1}{ms+b+\frac{k}{s}}.$$ (1)

**FIGURE 2:**
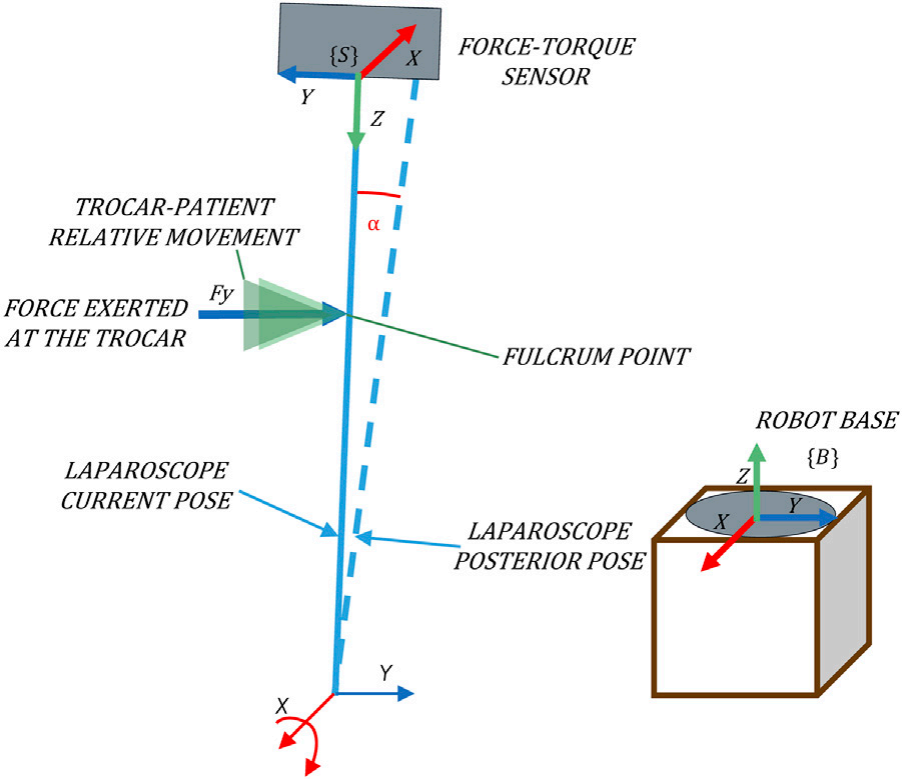
Pivoting movement as a consequence of forces exerted in the direction of the *Y*-axis of {B}.

The objective of an admittance control is to shape the mechanical admittance of a device such that it possesses desired characteristics. In this proposal, it is used to determine the angular velocity that minimizes the interaction forces. Figure 2 shows that the rotational velocity of the TCP that characterizes the pivoting motion is related to the forces exerted at the trocar. The exerted force $F_{y}^{B}$ would be avoided by a negative rotation in the *X*-axis of {B}. Analogously, if the force is exerted in the *X*-axis, $F_{x}^{B}$ , it can be avoided by a rotation around the *Y*-axis of {B}.

The mechanical admittance can be simplified so that it consists of only one damping. Eq. 2 describes this simplified admittance, which provides good results in terms of robustness and control accuracy despite its simplicity.

$$Y\left(s\right)=\frac{v}{F}=\frac{1}{b}.$$ (2)

By this model, we can determine a relationship between force and angular velocity that constrains the working space of the camera around the *X*- and *Y*-axes, defined as $\omega_{Ax}^{B}$ and $\omega_{Ax}^{B}$ at Eqs 3, 4, respectively, where the sub-index A stands for admittance. The gains applied to relate force and angular velocity, $k_{Ax}$ and $k_{Ay}$ , are experimentally calculated and are dependent on the environmental stiffness (Zeng and Hemami, 1997).

$$\omega_{Ax}^{B}=k_{Ax}F_{y}^{B},$$ (3)

$$\omega_{Ay}^{B}=k_{Ay}F_{x}^{B}.$$ (4)

For its implementation, a F/T sensor placed at the laparoscope’s coupling is used to determine the applied force at the trocar, taking {S} as a reference system (Figure 2). Note that to relate this force to the velocity using the admittance control, both variables must refer to the same system. The absolute reference system, which corresponds to the base of the robot {B} (Figure 2), is used. Hence, it is necessary to transform the force measurements to the absolute reference system {B}.

The aforementioned angular velocities allow the parameterization of the twist vector, ( $s_{A}^{B}$ ), which represents the linear and angular velocity of the TCP as shown in Eq. 5. This vector characterizes the pivoting motion of the tool that minimizes the interaction forces at the trocar, constraining the tool orientation and thus freeing the Cartesian control of the TCP.

$$s_{A}^{B}={\left({\left(v_{A},\omega_{A}\right)}^{B}\right)}^{T}={\left(0,0,0,\omega_{Ax}^{B},\omega_{Ay}^{B},0\right)}^{T}.$$ (5)

Forces are used to calculate the pivoting motion of the camera. Forces close to zero causes low pivoting speed, while higher order forces provoke higher response speeds. This strategy favours that the camera always tends to a resting position without aggressive movements, and consequently the forces exerted on the patient are minimized.

#### 2.2.2 Feedforward orientation control

The vector calculated at the TCP by the admittance control ( $s_{A}^{B}$ ) allows to minimize the forces exerted when the Cartesian velocity at the tool’s tip is zero. However, it is also convenient to maintain low contact forces once the camera is in motion to change the field of view. When there is a movement of the camera, the exclusive use of the admittance control would require a too elevated velocity or a high gain, causing oscillations and abrupt changes of direction, and therefore high forces on the trocar. This problem has been previously reported by Calanca et al. (2016) and may be solved with the addition of a position control loop that prevents large forces from being exerted, so a lower gain can be applied to the admittance control, avoiding an oscillatory behaviour.

**FIGURE 3:**
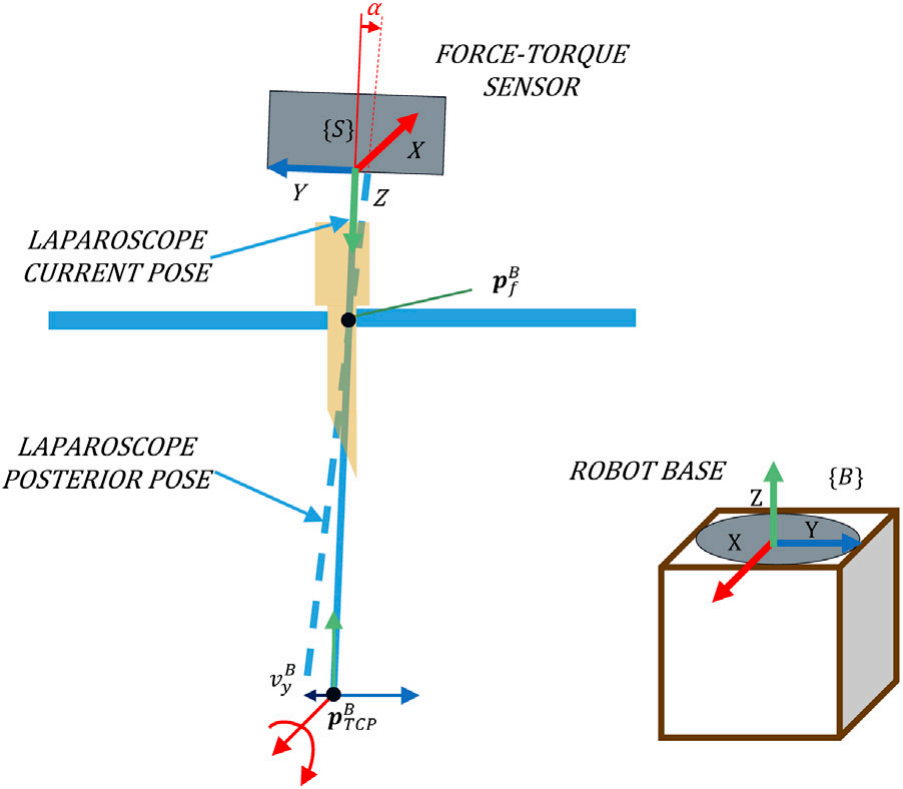
An adequate rotation related to the Cartesian velocity $v_{c}^{B}$ prevents exerting forces when the TCP of the laparoscope, or point $p_{TCP}^{B}$ , is displaced.

This position control loop can be implemented as a feedforward (ff). Figure 3 shows that it is possible to geometrically determine which rotation the tool should perform against the future Cartesian velocity of the TCP. The objective of this control is to limit the Cartesian velocity of the camera when Cartesian movement occur in the immediate future. The ff predictive action complements the admittance control, which minimizes the forces exerted in the present by pivoting, displacing the tool to a rest position as a consequence of the forces measured. Hence, this control ensures that the forces exerted are minimized without giving up the direct control of the TCP position.

For the calculation of the angular velocity, the ff controller needs to know the position of the camera point whose Cartesian velocity would be reduced due to this control. This point will be referred to hereinafter as the fulcrum position ( $p_{f}^{B}$ ), and it is the projection of the contact point between the laparoscope and the trocar at the laparoscope’s longitudinal axis. Eqs 6, 7 allow the calculation of the tool’s angular velocity at $p_{TCP}^{B}$ that keeps the camera immobile at the point $p_{f}^{B}$ . $d_{t}^{B}$ is the distance travelled between $p_{TCP}^{B}$ and $p_{f}^{B}$ in the time period ∆t . In the example of Figure 3, when displacing $p_{TCP}^{B}$ at a speed $v_{y}^{B}$ , the angle *α* of rotation that prevents forces around the *X*-axis is the difference between the angle formed by the vector $d_{t}^{B}$ in the YZ plane at the current and future instances. This calculation is performed in the YZ and XZ planes to determine the rotation using the *X*- and *Y*-axes, respectively, allowing to create the velocity vector of the ff control $s_{f}^{B}$ , as shown in Eq. 8.

$$\omega_{fx}^{B}=\frac{\alpha }{∆t}=\left(\frac{d_{ty}^{B}+v_{y}^{B}\cdot ∆t}{d_{tz}^{B}+v_{z}^{B}\cdot ∆t}-\frac{d_{ty}^{B}}{d_{tz}^{B}}\right)\cdot \frac{1}{∆t},$$ (6)

$$\omega_{fy}^{B}=-\left(\frac{d_{tx}^{B}+v_{x}^{B}\cdot ∆t}{d_{tz}^{B}+v_{z}^{B}\cdot ∆t}-\frac{d_{tx}^{B}}{d_{tz}^{B}}\right)\cdot \frac{1}{∆t},$$ (7)

$$s_{f}^{B}={\left({\left(v_{f},\omega_{f}\right)}^{B}\right)}^{T}={\left(0,0,0,\omega_{fx}^{B},\omega_{fy}^{B},0\right)}^{T}.$$ (8)

While the admittance control pivots the camera in response to the contact force, the ff control calculates the angle that the laparoscope must pivot in advance to maintain the fulcrum point at a low speed. In this way, the ff controller reduces the contact forces and ensures that the admittance control maintains its corrective functionality. The angular velocity vector calculated by the ff is added to the provided by the admittance control, thus allowing both preventive and reactive orientation controls even if the TCP is moving.

#### 2.2.3 Movement in the released degrees of freedom

**FIGURE 4:**
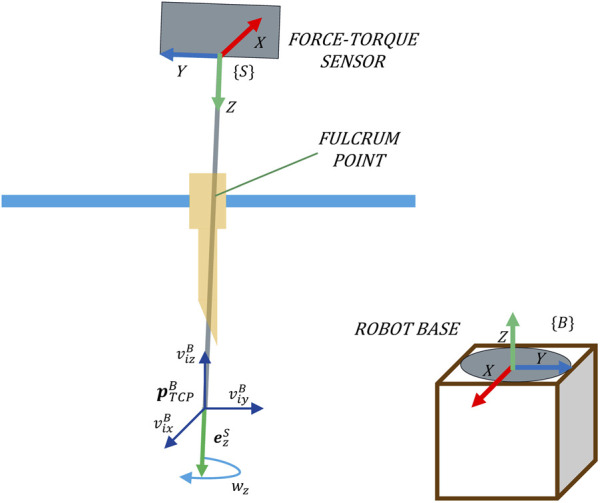
Four DoFs released at the TCP by the proposed force-based pivoting motion control.

The proposed force-based pivoting motion control liberates the TCP movement, characterized by velocity $v_{ix}^{B},v_{iy}^{B},v_{iz}^{B}$ . Movement is also released around the longitudinal axis of the laparoscope $e_{z}^{s}$ characterized by the rotation velocity $w_{z}$ , since this will not cause any contact forces with the patient. This allows to transform the four DoFs available in the RCM approach (Figure 1) into 3 DoFs of Cartesian motion and 1 DoF of rotation (Figure 4).

The twist velocity vector $s_{i}^{B}$ , which defines the four DoFs released at the TCP referred to the robot base{B}, is described in Eq. 7a. $s_{i}^{B}$ is referred to the system {B}, and consequently, the Cartesian velocities and the angular velocity must be also referred to{B}. Hence, the angular velocity $w_{z}$ of the tool through its longitudinal axis $e_{z}^{S}$ is transformed to the system {B} using the rotation matrix $R_{S}^{B}$ .

$$s_{i}^{B}={\left({\left(v_{i},\omega_{i}\right)}^{B}\right)}^{T}={\left(v_{ix}^{B},v_{iy}^{B},v_{iz}^{B},{w_{z}e_{z}^{S}R}_{S}^{B}\right)}^{T}.$$ (7a)

The reference velocity, considering the four DoFs of the TCP, can be defined without worrying about the exerted forces since they would be minimized by the control.

### 2.3 Complete force-based pivoting motion control strategy

**FIGURE 5:**
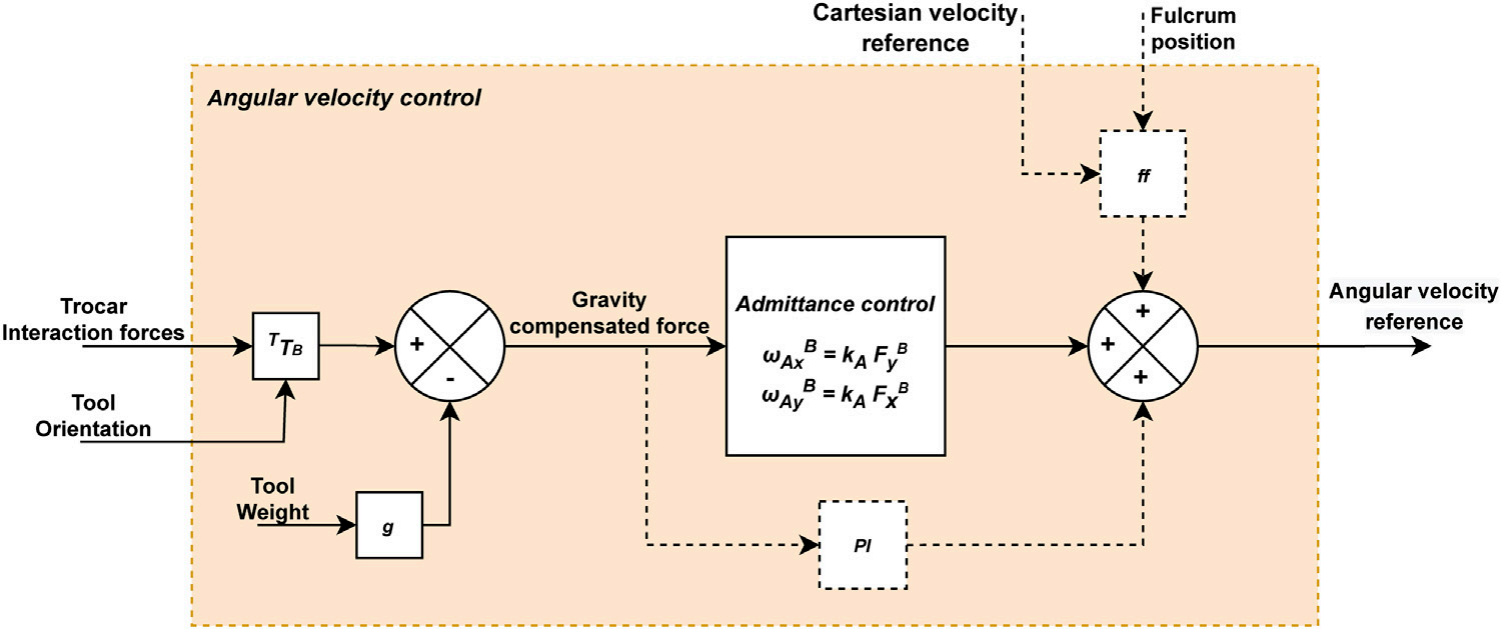
Diagram of the force-based angular velocity control for the laparoscope. The dashed lines connect elements that are only activated by moving the laparoscope’s tip.

The complete force-based pivoting motion control is shown in Figure 5. This control strategy is the result of the sum of the admittance and ff controls described previously. A PI controller is also included, although it could be omitted in case the exact position of the fulcrum point $p_{f}^{B}$ is known. The PI and the ff controllers, shown with dashed lines in Figure 5, should only act when the camera is in motion.

PI complements the predictive action of the ff when the position of the fulcrum point $p_{f}^{B}$ is not accurate, since the ff control would not completely eliminate the forces exerted on the trocar during the movement. This would cause a stationary error during Cartesian $p_{TCP}^{B}$ movement that the admittance control could not correct. Therefore, the inclusion of the PI integral action compensates the imperfection of the ff controller associated with a poor fulcrum point estimation. Due to the dynamic nature of the system, where continuous trocar displacements may occur due to physiological and camera movements, we proposed an operation mode for this PI control since an over-action would affect the quality of the control strategy. The PI control is only active when the camera is in motion, and therefore, it is configured to reset after each change of direction or stop of $p_{TCP}^{B}$ . In addition, the PI control must be adjusted with a low gain and an appropriate control frequency in such a way that past states do not affect the present control, or in other words, so that it can be determined whether the stationary error is still present and to prevent past forces already compensated to affect the control. In the experimental results, control parameters and its performance are analysed.

**FIGURE 6:**
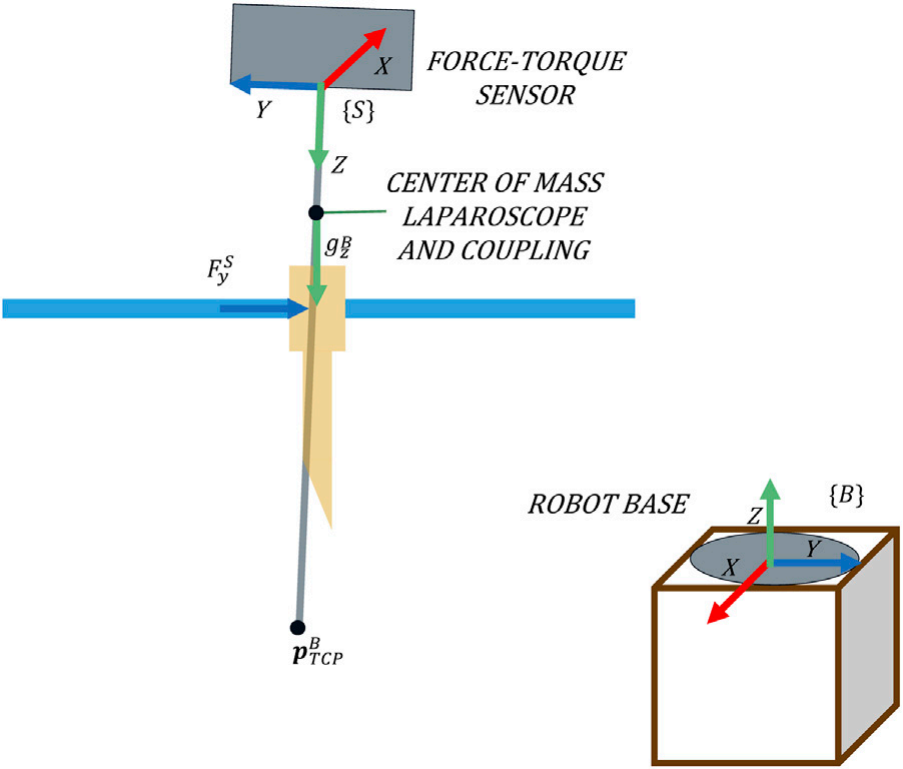
Forces measured on the trocar and their reference systems.

To sum up, interaction forces are reduced due to the pivoting motion provided by the admittance control. Additionally, the ff control anticipates the laparoscope’s motion to prevent the appearance of forces due to this motion, and the PI control eliminates errors that have not been corrected in the immediate past. For this purpose, the forces exerted at the trocar point, once gravity has been compensated, are inputted to the admittance control and the PI, while the ff control requires the current and desired (future) positions of the TCP ( $p_{TCP}^{B}$ ) and the current position of the fulcrum ( $p_{f}^{B}$ ). Additionally, not only the force measurements and orientation of the tool but also the weight of the tool is needed to calculate the gravity-compensated force. As previously mentioned, all control variables are expressed in the same reference system associated with the robot base {B}. Hence, the gravity can be directly compensated (Figure 6).

### 2.4 Setup platform

**FIGURE 7:**
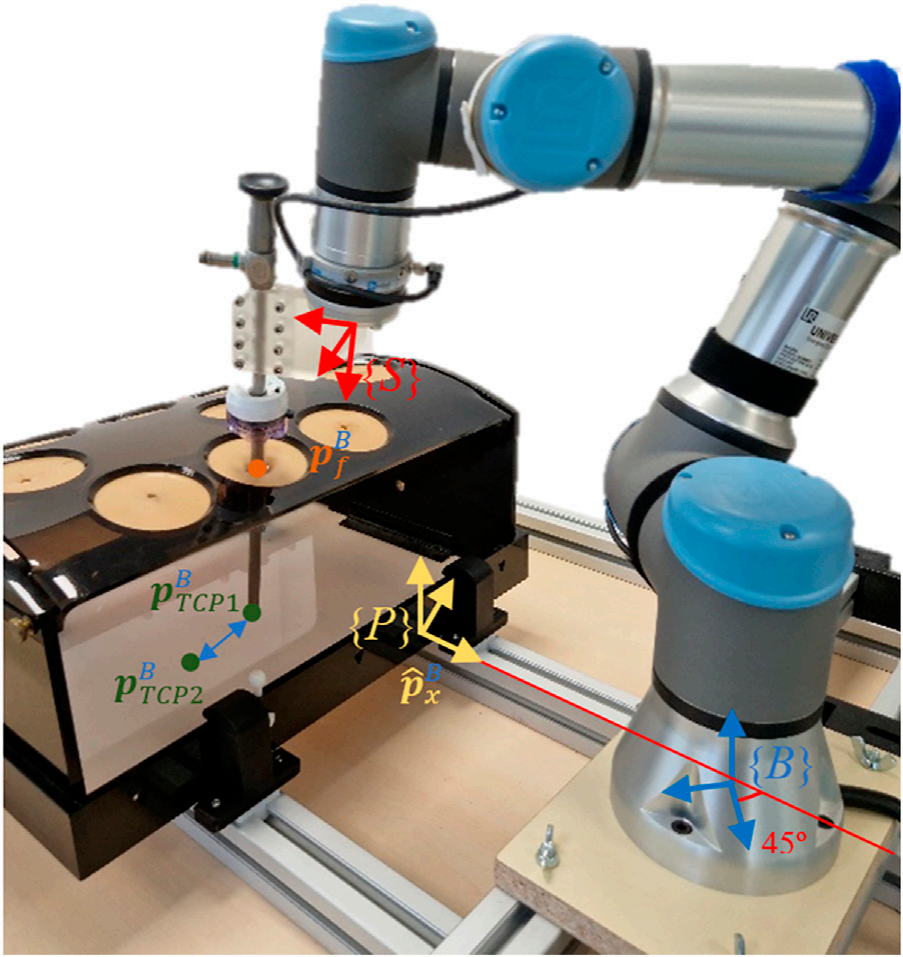
Experimental setup mainly consisting of the robot, F/T sensor, and pelvitrainer.

The setup platform used to test the performance of the presented force-based control strategy is shown in Figure 7. The experimental platform mainly consists of a 6-DoF UR3e robot (Universal Robots, Denmark), a Hex-H F/T sensor (OnRobot A/S, Denmark), and a SZABO-BERCI-SACKIER pelvitrainer (KARL STORZ SE & Co. KG, Germany). The reference systems which are required by the theoretical proposal {B} and {S} are located at the base of the UR3e Robot and the F/T sensor, respectively. A new reference system {P} is attached to the pelvitrainer (Figure 7).

The pelvitrainer recreates the mobility restrictions in laparoscopic surgeries, due to the rigidity of its orifices, which mimic those of a real abdomen. To test the safety of the proposed control, a 3D-printed PLA rails were arranged for the pelvitrainer to move along ${\hat{p}}_{x}^{B}$ axes. In this way, it is possible to test the control response to small movements that mimic the patient’s physiological movement, as well as larger movements, which simulates accidents that might happen in collaborative environments.

The F/T sensor, used to measure the interaction forces at the trocar, has an accuracy of 0.2 N in the XY plane of {S}, and its maximum frequency sample is 500 Hz. The surgical instrument is introduced inside the abdomen through 10-mm and 12-mm ENDOPATH XCEL^®^ trocars. A Storz Telecam One-Chip Camera Head 20212030 is used which is integrated in a HOPKINS telescope 0° with 10 mm diameter. This cylindrical device is inserted into the trocar and in turn into the interior of the pelvitrainer. It has been coupled to the robot by means of a rigid resin coupling tool of our own manufacture, in order to avoid unwanted plastic deformation and inaccuracy in the positioning of the TCP.

### 2.5 Control system implementation

**FIGURE 8:**
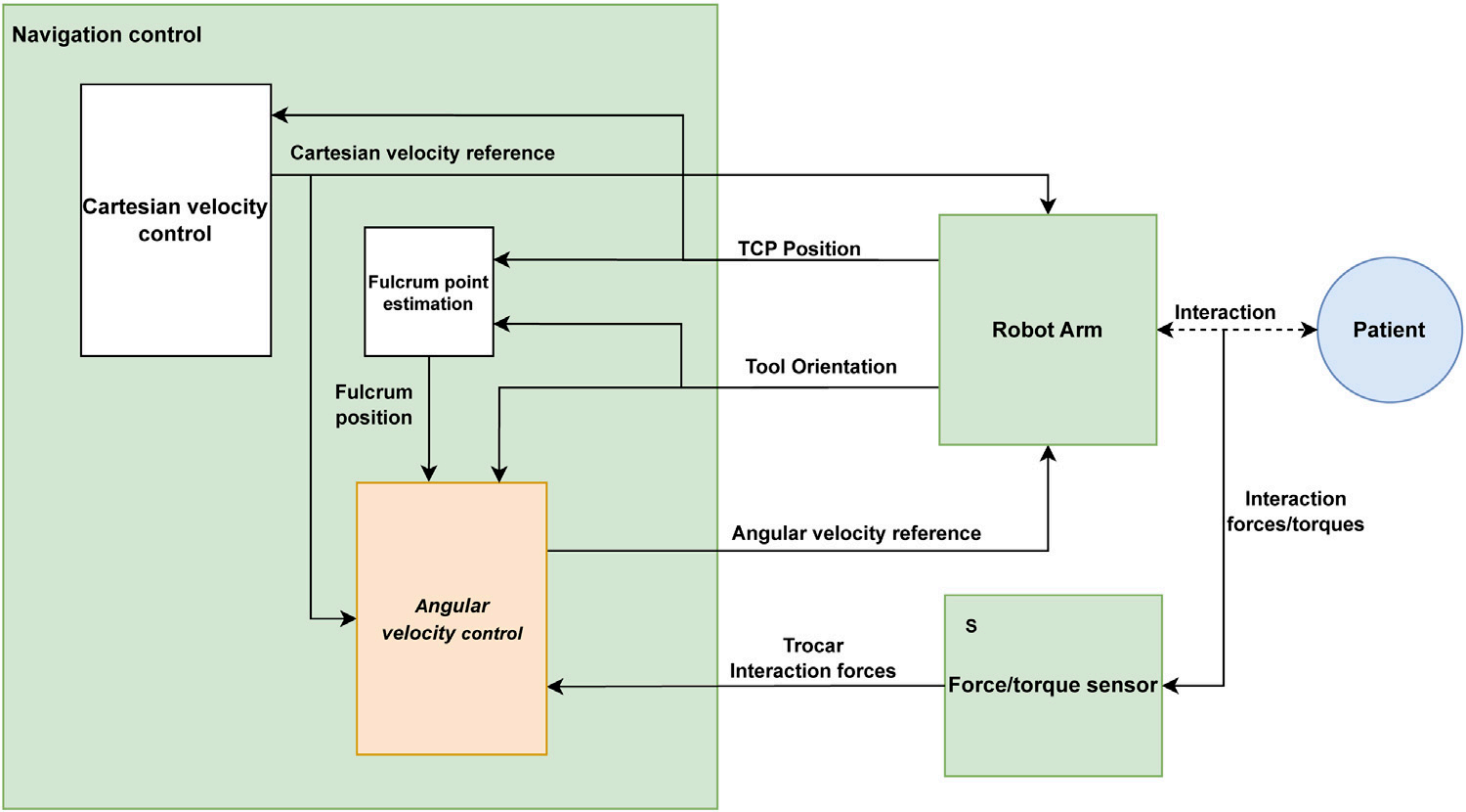
ROS implementation diagram of the robot control.

The proposed control architecture is implemented using ROS (Robot Operating System) and is illustrated in Figure 8. The green blocks correspond to ROS nodes, being three main blocks: the navigation control, the UR3e robot, and the HEX F/T sensor. The navigation control is subdivided into three specific tasks. The Cartesian velocity control and the fulcrum point estimation (white blocks in Figure 8) can be freely implemented and are not detailed in this manuscript. For the experimental tests, the reference Cartesian velocity has been defined according to the experimental goals. Additionally, experimental results showed that it is not necessary to provide the precise fulcrum location for the control strategy to work correctly. In fact, a 5 cm height error provides an appropriate response, which improves if the point is determined accurately.

The angular velocity control (orange block in Figure 8) is the one responsible of controlling the TCP’s angular velocity under the principles presented in this paper. A 4-point average filter is applied to the force measurements to reduce the noise, resulting in a filtered force with a frequency of 125 Hz. This filtered force combined with the reference Cartesian velocity of the TCP, fulcrum position, and tool orientation is used to calculate the reference angular velocity by the angular velocity control.

The communication between the ROS nodes is carried out by means of ROS topics, through the subscriber–publisher relationship that they maintain. The SpeedL command, provided by Universal Robots on 0F^1^URScript, is sent to the UR3e robot with the desired velocity vector of the $p_{TCP}^{B}$ point, generated on the navigation control block, as shown in Figure 8. This command requests the robot every 8 ms to control the absolute velocity of a point in real time in a fluent way.

## 3 Results

The proposed force-based pivoting motion control has been tested using the experimental setup shown in Figure 7. First, the pivoting motion generated by the admittance control has been evaluated as a method to control the forces exerted on the trocar. Second, the effectiveness of the ff and PI together with the admittance control has also been tested to avoid exerting forces on the patient due to the Cartesian motion of the TCP. Third, the performance of the proposed control has been tested against errors in the estimation of the fulcrum position, to validate the robustness of the control in real environments. The detection and minimization of risks in the event of accidents have been tested. Finally, the ability of the control to maintain the FoV of the camera at a fixed point during pelvitrainer movements has also been evaluated.

### 3.1 Admittance control

**FIGURE 9:**
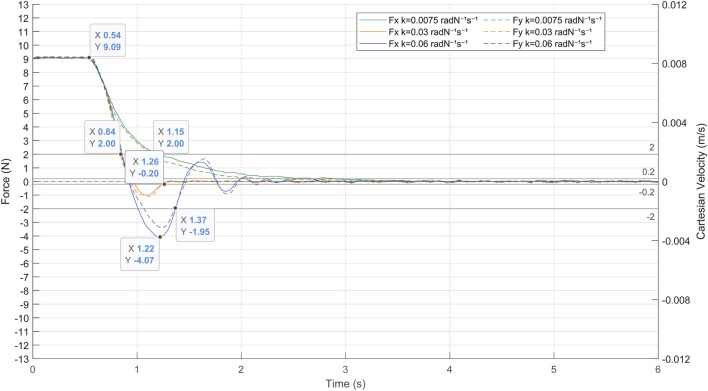
Response of the admittance control to a contact force of 9 N between the laparoscope and the pelvitrainer, characterized by a radial stiffness of 3 N/mm.

The first experiment aimed to determinate the gain of the admittance control by evaluating its response to the contact forces produced between the laparoscope and the patient. The patient’s abdomen was simulated by a pelvitrainer characterized by a radial stiffness of 3 N/mm, which is a representative value of the human tissue. In this experiment, the pelvitrainer was moved in the ${\hat{p}}_{x}^{B}$ direction (Figure 7), resulting in a movement of 3 mm in the *X*- and *Y*-axes of {B}. This displacement caused a constant force of 9 N in each axis. The response of the system to this external force is shown in Figure 9. The force on the *X*-axis is shown as a solid line, while the force on the *Y*-axis is represented with a dashed line. The figure shows that once the admittance control is activated, the contact forces of 9 N are minimized by the pivoting motion of the TCP.

The gains $k_{Ax}$ and $k_{Ay}$ of the admittance control are experimentally determined. Both gains are equal since the system should respond in the same manner on both axes. Therefore, three experimental tests were carried out with the following gain values: 0.03, 0.06 and 0.075 radN^-1^s^-1^.

There is also a clear need to avoid high or low gains since the system loses efficiency in both cases. A high gain (blue line in Figure 9) provokes undesired and unsafe oscillations, while a low gain (green line in Figure 9) causes an overdamped response, which increases the time response of the system. In the case of a gain of 0.03 radN^-1^s^-1^ (orange line in Figure 9), the system stabilizes quickly, not exceeding ±0.2 N again at 0.2 s after the over-peak and 0.7 s after the start of the pivoting. It is noteworthy that, with this gain, the system takes 0.32 s to reduce the exerted forces of 2 N, a threshold at which the risk can be considered avoided.

It has been proved that the system provides an adequate response to high forces and has therefore been validated to be effective in minimizing the contact forces produced by any movement of the patient, including slight physiological movements. The following experimental tests are carried out with an admittance control determined in this experiment at 0.03 radN^-1^s^-^1. Note that the mechanical characteristics of the pelvitrainer, such as the stiffness, may slightly differ from a patient’s abdominal wall, so the gain might require some fine-tuning.

### 3.2 Force-based pivoting motion control

**FIGURE 10:**
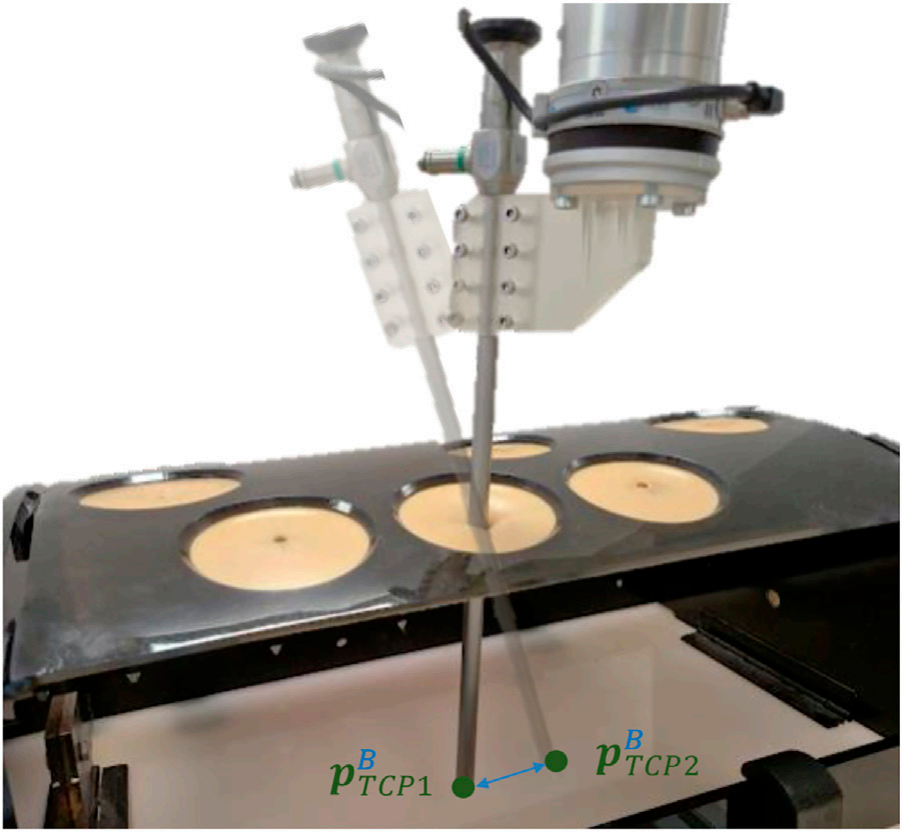
Linear movement performed to test the ff pivoting control for the Cartesian movement of the TCP.

**FIGURE 11:**
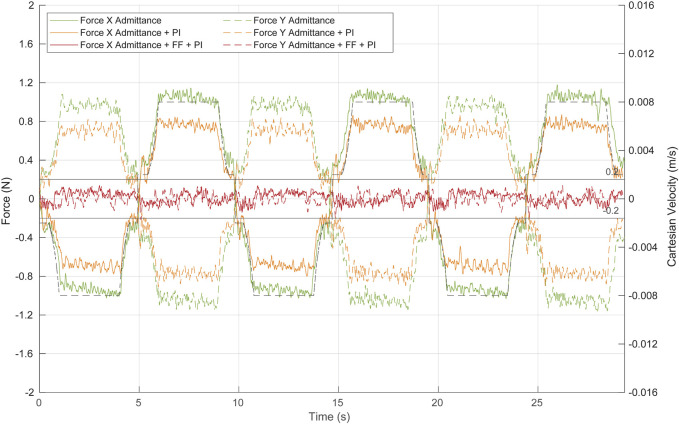
Force at the trocar during Cartesian movement of the TCP by the combination of the different control algorithms. Green, admittance control; orange, admittance control and PI; red, complete control strategy: admittance control, PI, and ff. Continuous lines represent forces at the *X*-axis, while dashed lines show forces at the *Y*-axis. The dashed black line represents the TCP Cartesian speed at the *Y*-axis.

The performance of the complete force-based pivoting control to minimize the forces exerted by the camera during its movement has also been evaluated. The admittance control in combination with the action of the PI and ff controls is tested against repetitive linear movements of the TCP between $p_{TCP1}^{B}$ and $p_{TCP2}^{B}$ , producing the motion shown in Figure 10. The displacement of the TCP is 3 cm in the *X*- and *Y*-axes of {B}, without height variation, introducing the laparoscope to a depth of 15 cm inside the pelvitrainer. In the following experiments, the reference Cartesian velocity is 8 mm/s in both axes, decreasing linearly as the TCP approaches the target zone, to observe if there were any changes in the behaviour at lower speeds. Figure 11 represents the reference Cartesian velocity in Y (black dashed line) to show the relationship between the TCP velocity in *Y*-axis and the force appearing in this axis in the different tests. The X Cartesian velocity of the TCP has been omitted to simplify the graph, as it is opposite to the Y velocity, producing an X force also opposite to the Y force.

The first test (green line in Figure 11) aimed to evaluate the performance of the admittance control during these linear movements of the TCP. Although the admittance controller operates alone (neither ff nor PI has been implemented), the system response is closed to be considered safe, narrowly exceeding ±1 N as the maximum force. The contact force decreases during deceleration of the TCP displacement, indicating that once the ff is implemented, it will effectively reduce the force exerted at the contact point, even if its position is not accurately estimated.

The second test (orange line in Figure 11) evaluated the response of the system to the TCP displacement, when the system is composed of the admittance control and the PI control. The PI, which requires current and past force measurements, is characterized by a low integral gain of 0.00015 rad/(s∙N) and a frequency of 100 Hz. Note that the PI control has been activated only during the TCP point. A low value of this gain allows to reduce part of the stationary error during the Cartesian motion of the TCP, while a high value produces oscillations. The inclusion of the PI controller improves the response of the system without implementing yet any kind of position control. Figure 11 (orange line) shows that the forces exerted during the TCP displacement never surpass ±0.9 N, and therefore, the control can be already considered safe.

The last test (red line in Figure 11) was performed with the complete angular velocity control, which consisted of the combination of the admittance, PI, and ff controllers. The ff needs the fulcrum position and the reference Cartesian velocity to provide a predictive position control. In this experiment, the depth of the fulcrum point was manually measured. During 95% of the test time, the forces in the *X*- and *Y*-axes exerted by the camera during its displacement were in the range [-0.13 N 0.13 N]. Therefore, the predictive action of the ff reduces significantly the force exerted compared to the previous tests.

### 3.3 Force-based pivoting motion control with uncertainty at the trocar

The performance of the complete angular velocity control has been previously evaluated against forces exerted by the camera motion. However, the fulcrum position, which is required by the ff controller, may be uncertain or vary during surgery due to physiological movements of the patient, such as breathing. Hence, in this section, we aimed to repeat the previous test but including a depth error in the fulcrum position.

**FIGURE 12:**
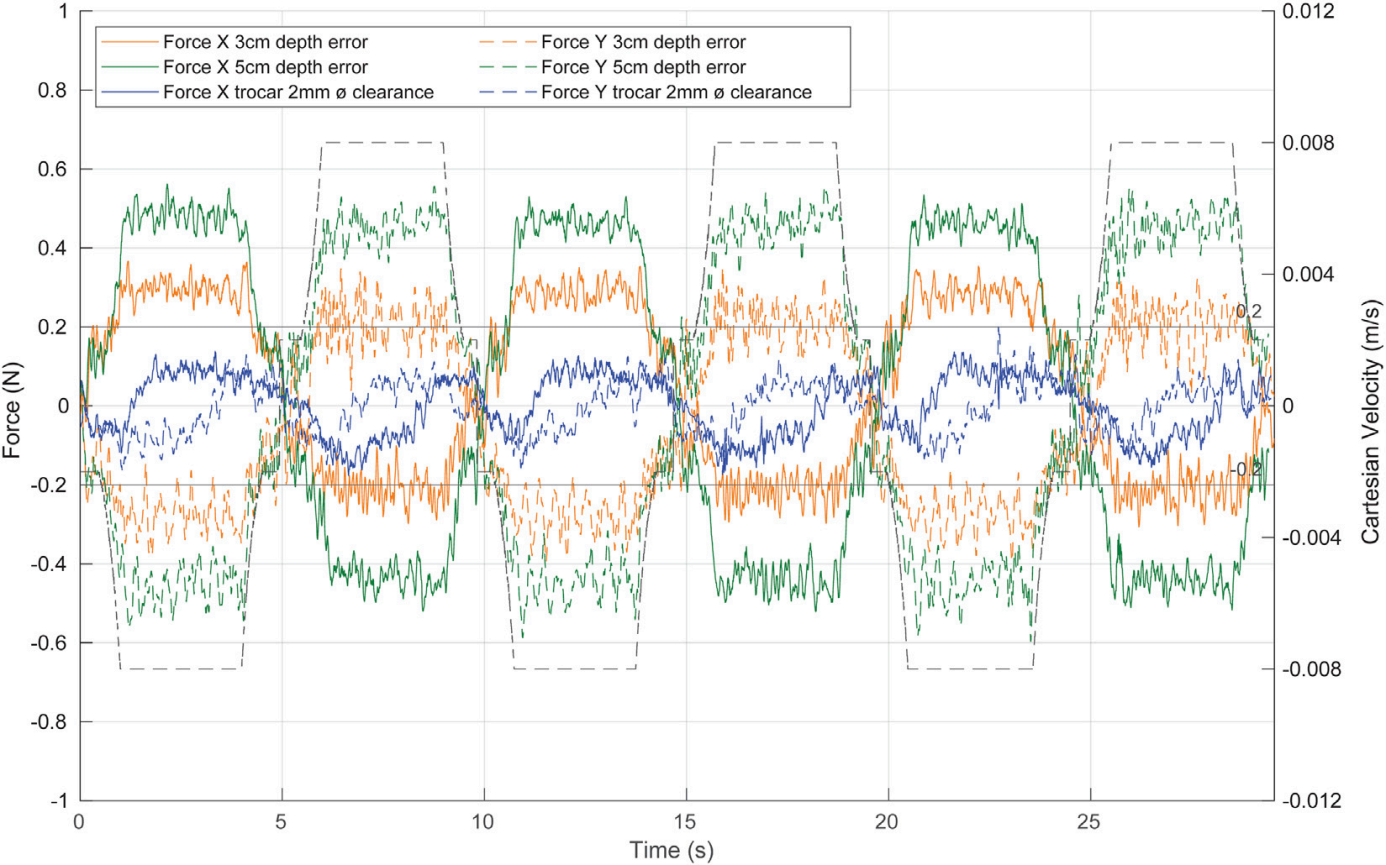
System response to repetitive linear movement of the TCP in different uncertainty scenarios. Green, with a fulcrum height error of 5 cm; orange, with a fulcrum height error of 3 cm; blue, with a trocar clearance of 2 mm. The dashed black line represents the TCP Cartesian speed.

Figure 12 shows the response of the system to repetitive linear movement of the TCP (dashed black line) with a fulcrum height error of 3 cm (orange line) and 5 cm (green line). The effectiveness of the ff decreases compared to the analogue results without the height error (Figure 11, red line). However, the complete angular velocity control outperformed the admittance and PI controls (Figure 11, orange line) even with an erroneous estimation of the fulcrum position.

The predictive action of the angular velocity ff control limits the speed of the laparoscope at the contact point, improving the effectiveness of the admittance control in reducing the contact force caused by the movement of the tool. In fact, the contact forces in the *X*- and *Y*-axes are reduced to a range [-0.6 N 0.6 N] even with a 5 cm height error (Figure 12, green line). With a height error of 3 cm (Figure 12, orange line), the exerted forces are within the [-0.4 N 0.4 N] range. If the entry point for the laparoscope is taken as a $p_{fz}^{B}$ approximation, the error would always be less than 3 cm which can be considered the most unfavourable case.

Additionally, to test the performance of the control with less contact information, a trocar with a clearance of 2 mm is used. The system reacts more slowly than the previous tests (Figure 12, blue line), as the trocar tolerance generates a less restrictive scenario. We can therefore assure the performance of the control in various ranges of possible situations and forces applied.

### 3.4 Test against accidents

If the TCP of the camera is immobile, the admittance control is the only control that is operative, and it will produce a pivoting motion to minimize any contact force. If the TCP is moving, the ff is activated so its predictive action reduces the movement at the contact point, ensuring a good response of the admittance control. It has been tested that the proposed force-based pivoting motion control efficiently reduces the contact forces at any time.

Additionally, a safety mode has been implemented to detect force-related abnormal conditions and hence drastically reduce any risk situation to the patient. If the force exceeds a predetermined threshold, the safety mode immobilises the laparoscope’s tip. The threshold is set to 2 N in the horizontal plane, which is considered the safe force limit. The reference Cartesian velocity of the tip is automatically set to zero, while the admittance control would reposition the camera by pivoting, thus minimizing the interaction force.

Although for this implementation the safety mode immobilizes the laparoscope’s tip, another alternative would be to provide a reference Cartesian velocity such that it counteracts the excessive force. This alternative, although it requires a more complex implementation of the Cartesian velocity control, would have a faster response time since the admittance control is complemented by this reference motion.

**FIGURE 13:**
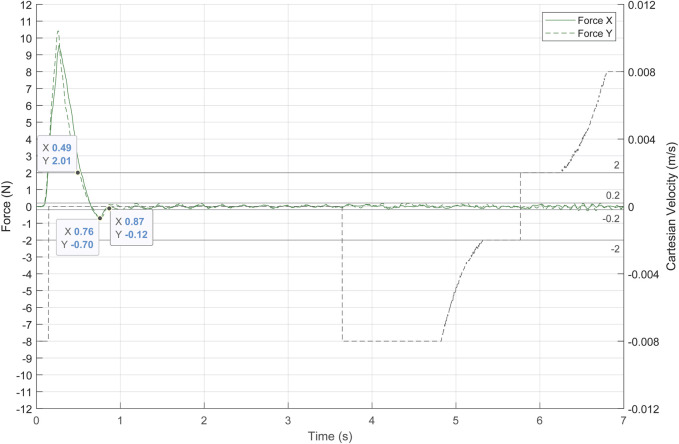
System response to an accident, simulated by a fast movement of the pelvitrainer. The dashed black line represents the TCP Cartesian speed, and the green lines represent the contact forces.

The following experimental test has been performed with the safe mode that sets the reference speed to zero when the force threshold is exceeded since it is the simplest and most unfavourable case. The response of the system to a dangerous situation is shown in Figure 13. At the beginning, the laparoscope performed the linear movement of the previous tests at a depth of 15 cm. Then, an accident was recreated by making the UR3e robot to displace the pelvitrainer in the ${\hat{p}}_{x}^{B}$ direction of 1 cm at a speed of 10 m/s in each *X*- and *Y*-axis of {B}. Therefore, a peak force of about 30 N should have appeared with the displacement and the elastic constant of the pelvitrainer (3 N/mm). However, the pivoting damped the impact force. Considering the stiffness of the pelvitrainer, which is 3 N/mm, and the displacement in this test, which is 10 mm, the pivoting movement helps to cushion the initial impact. The peak force is reduced from an expected value of 30 N to 10.34 N. After the initial peak, the force returns to a safe value of 2N within 0.24 s. The over-peak occurred at 0.51 s from the peak with a value of 0.7 N, a force that can be considered completely safe. After 0.06 s, the force decreased to the 0.2 N range, where forces can be considered negligible. When detecting excessive forces, the safe mode set the reference Cartesian velocities to zero. After a considerable time from the accident detection, the reference Cartesian velocity of the TCP was reactivated.

### 3.5 Capability to maintain the field of view

**FIGURE 14:**
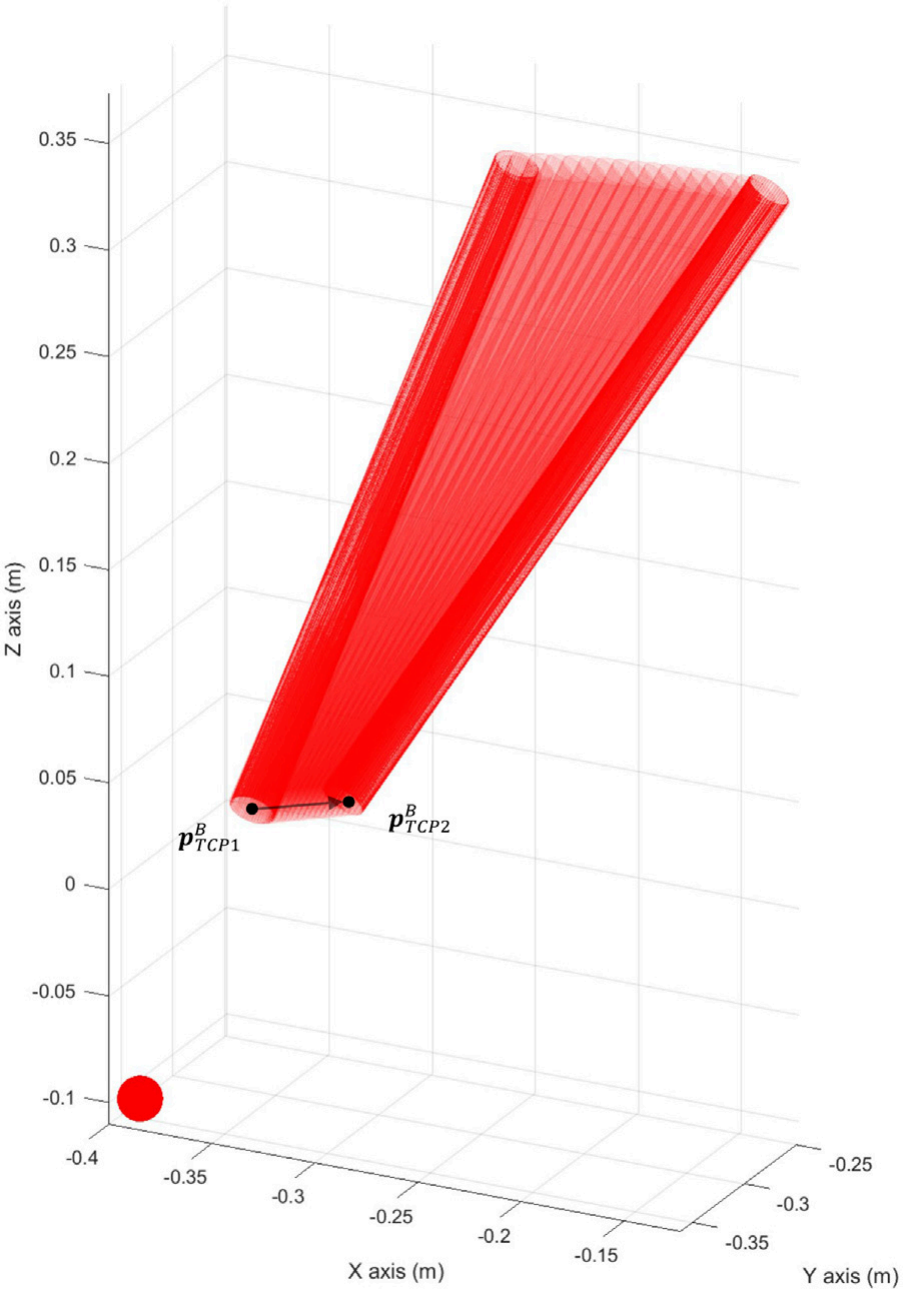
Camera keeping track of the target point $p_{r}^{B}$ while the pelvitrainer is in motion.

The aim of the proposed control strategy is to allow keeping the FoV of the camera using the four DoFs while minimizing the exerted forces. The laparoscope was inserted into the trocar up to a depth of 10 cm, and a target point ( $p_{r}^{B}$ ) was set for the camera to track during the experiment. $p_{r}^{B}$ was defined as the intersection between the initial longitudinal direction of the laparoscope and the plane Z^B^ = −0.1 m (Figure 14). The pelvitrainer was moved 5 cm at 3 mm/s in the *X*- and *Y*-axes of {B}, which coincides with the ${\hat{p}}_{x}^{B}$ direction (Figure 7). The reference Cartesian velocity was set so that the laparoscope’s TCP moves to keep track of $p_{r}^{B}$ . Figure 14 shows how the camera keeps track of the target point and hence maintaining the desired FoV.

**FIGURE 15:**
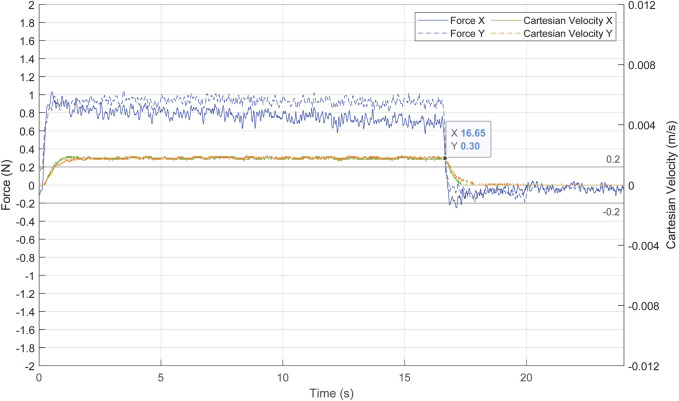
Forces exerted at the trocar in blue and Cartesian velocity of the TCP in green and orange.

The force exerted at the trocar and the actual Cartesian velocity of the TCP is displayed in Figure 15. The forces exerted were at a low risk level, not exceeding 1.1 N. During the movement of the pelvitrainer, the camera also moved to keep pointing to $p_{r}^{B}$ , while the force-based control generated the pivoting motion to minimize the contact forces. Once the movement of the pelvitrainer stopped (16.68 s), the laparoscope kept moving to maintain the FoV. At this point, forces decreased rapidly even while the laparoscope was still displacing, since the PI and ff prevented exerting forces caused by the laparoscope’s tip movement.

## 4 Discussion

The proposed control strategy, which considers the trocar as a non-static element, is closely related to the proposal by Krupa et al. (2000) since both control approaches have been designed under the same theoretical considerations. The experimental results presented by Krupa showed that the control they proposed reduced the contact forces at the trocar to ±2 N. However, that force is relatively high since it is equal to the maximum force limit that we have considered safe. These results may be caused by the low refresh rate of the F/T sensor used, which was 50 Hz. Despite the later widespread use of the RCM approach, we consider that the approach proposed by Krupa with proper sensing technology has a longer run.

The experimental results presented in this article verified that the developed strategy based on the theoretical proposal using a current F/T sensor minimizes the contact forces and hence makes the laparoscopy surgery safer. Among these improvements, the positioning of the robot prior to the intervention is facilitated, as less precision is required, and no stationary forces are exerted on the patient. This is the reason why we would like to rethink the use of force-based controls and to highlight Krupa’s work.

In addition to the technological gap, there is also a difference in the mobility principles in both proposals. While Krupa’s proposal and other mobile RCM-based solutions control the position of the RCM, our proposal controls the velocity of the tip of the camera, with the inherent advantage that there is no need to know the precise position of the fulcrum. By the nature of the pivoting motion, once the force is known, it is possible to rotate the tip of the camera until the force disappears without knowing the exact fulcrum position. Therefore, the pivoting motion for repositioning the laparoscope is an appropriate strategy to control the forces at the trocar.

The effectiveness of the proposed force-based pivoting motion control has been verified when the tip of the camera, or pivot point, is static. In the presence of an external force of 9 N, the control takes 0.7 s to minimize the force to ±0.2 N and only needs 0.32 s to reduce the force to 2 N. However, when the pivot point is moved, forces are exerted on the patient. Therefore, a predictive ff controller is activated when there is motion in order to anticipate it and rotate the camera around its tip. In this way, the forces exerted by the tool are limited in advance for the force-based control to function as effectively as it does when the tip remains static.

Although the mobile RCM and our proposed pivoting motion control strategies have a similar goal, the latter has the advantages that there is no need either to determine the exact fulcrum position or to displace the complete laparoscope to minimize the contact forces (Marinho et al., 2016). A mobile RCM that is designed to maintain control of the laparoscope tip would respond with a pivoting motion. This is equivalent to the control system proposed in this manuscript. Our approach decouples the camera tip position control from the orientation control that minimizes the contact forces in the background. This makes it possible to not lose sight of the tracked area even when physiological movements occur. This implies the possibility of maintaining the FoV, which was also tested.

This specific mobility strategy for force-feedback robots is ideal for camera handling, due to the precise control of the TCP position. It allows to control where the camera is pointing at any given moment without a precise method of determining the trocar position. We foresee that the proposed mobility strategy can be applicable to the robotic handling of other types of laparoscopic tools. For the camera, only contact forces at the trocar are exerted, but during the handling of other tools, tissue contact forces also occur. Therefore, it will be necessary to differentiate the contact forces on the trocar from those applied on the tool. This has been discussed in the literature as an inherent problem in this environment, forcing the implementation of the mobility constraints of the RCM to directly minimize the forces on the trocar. However, we believe that if contact forces are distinguished, this mobility strategy would maintain its characteristics and benefits for tool handling while allowing haptic feedback for the surgeon.

## 5 Conclusion

This paper presents a force-based pivoting motion control strategy for a collaborative robotic system for laparoscopic surgery. Its performance was evaluated in a set of experiments that showed a drastic reduction of the contact force that the instrument exerts to the abdominal walls, even in the presence of motion. It can be therefore concluded that this approach ensures safety during the laparoscopy surgery. Additionally, this force-based control strategy has some advantages over the mobile RCM approach: there is no need to know the exact position of the trocar and liberates the motion of the camera tip. Therefore, the camera can point to any location of interest during surgery since the real environment constrains dynamically the camera orientation.

It is important to point out that the performed experiments have been conceived as a proof of concept. They were designed to evaluate the performance of the force-based control strategy for minimizing forces both against patient movements and camera movements. This allowed to define the scope of the method, and future experiments should be carried out based on scenarios closer to real surgery conditions (i.e., replacing the pelvitrainer with a dead or living animals and human body donors).

## Data Availability

The raw data supporting the conclusion of this article will be made available by the authors, without undue reservation.
